# The Pricing of Breakthrough Drugs: Theory and Policy Implications

**DOI:** 10.1371/journal.pone.0113894

**Published:** 2014-11-25

**Authors:** Moshe Levy, Adi Rizansky Nir

**Affiliations:** School of Business, The Hebrew University, Jerusalem, Israel; Tohoku University, Japan

## Abstract

Pharmaceutical sales exceed $850 billion a year, of which 84% are accounted for by brand drugs. Drug prices are the focus of an ongoing heated debate. While some argue that pharmaceutical companies exploit monopolistic power granted by patent protection to set prices that are “too high”, others claim that these prices are necessary to motivate the high R&D investments required in the pharmaceutical industry. This paper employs a recently documented utility function of health and wealth to derive the theoretically optimal pricing of monopolistic breakthrough drugs. This model provides a framework for a quantitative discussion of drug price regulation. We show that mild price regulation can substantially increase consumer surplus and the number of patients who purchase the drug, while having only a marginal effect on the revenues of the pharmaceutical company.

## Introduction

Pharmaceutical sales have grown dramatically over the last decade, from $365 billion in 2000, to $837 billion in 2009, and they are expected to exceed $1.1 trillion by 2015 [1]. The price of new drugs has also been rising over time [2]. 84% of drug sales are accounted for by brand drugs. This trend has led to a heated debate about drug price regulation. Critics of the pharmaceutical industry claim that pharmaceutical companies, that benefit from public investment in basic research, price brand drugs essentially monopolistically, protected by patent rights, leading to prices that are “too high”, especially in the U.S. While most countries regulate drug prices, either directly (e.g. France and Italy), or indirectly (e.g. U.K., Germany and Japan), the U.S. does not [3]. Indeed, drug prices are on average substantially higher in the U.S. than in the rest of the World. According to U.S. Department of Commerce International Trade Administration report [4], prices of patented drugs in OECD countries are 18%–67% lower than their U.S. prices (depending on the country of comparison). Thus, it is also argued that U.S. patients subsidize patients in the rest of the World. On the other hand, proponents of the pharmaceutical industry argue that drug prices reflect the very high R&D costs in the pharmaceutical industry, estimated at over $800 million per drug [5], and the low success probability, and that any price regulation will stifle innovation.

Clearly, both sides of this debate seem to have a point. In order to reach a conclusion regarding the question of drug price regulation, one must estimate its effects in a quantitative fashion. Namely, the main questions to be addressed are: what are the effects of drug price regulation on the revenues of the pharmaceutical company? What is the effect on the number of patients who will purchase the drug, and on the consumer surplus? In order to answer these questions one should estimate the demand function for the drug, as a function of its health benefits and its price. The standard economic framework for analyzing this demand function is the expected utility maximization framework [6]. In the context of health economics, well-being, or “utility”, is a function of both health and wealth (or consumption). Formally, 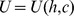 where *c* denotes wealth (or consumption, in a multi-period setting), and *h* denotes health on a scale of 0 (death) to 1 (perfect health). Out of all feasible 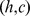 combinations, the individual choses the one that maximizes her utility. Thus, the shape of the utility function *U* reflects the individual's health-wealth tradeoff preferences, and plays a central role in understanding optimal drug pricing. Note that though individuals may not even be aware of the concept of expected utility maximization, they still may make choices “as if” they are utility maximizers [7].

Several studies have shown that the marginal utility of wealth increases with health [8]–[10]. Levy and Rizansky [11] investigate the utility of health and wealth by interviewing cancer and diabetes patients about their health-wealth tradeoffs. They find that the utility function
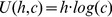
(1)provides a very good description of individuals' preferences. Here we employ this recently documented utility function to derive the demand for a drug as a function of its price, the health improvement it provides, and the patient's wealth. This demand function is the basis for analyzing the optimal monopolistic price from the point of view of the pharmaceutical company, and the effects of price regulation on the company's revenues and on patients' welfare.

The paper is organized as follows. In the next section we derive theoretical results regarding optimal monopolistic pricing. Then, we discuss the implications of the optimal monopolistic pricing to the debate over drug price regulation. We quantify the loss of revenue to the pharmaceutical company and the increase in patient welfare resulting from price regulation. We find that mild price regulation implies only a marginal effect on revenues yet leads to a large increase in the number of patients who use the drug, and to a substantial increase in consumer surplus. We conclude with a discussion of policy implications.

## Optimal Monopolistic Pricing

Consider a new breakthrough drug that offers a health improvement from 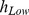 to 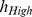, where *h* denotes the health level on a scale of 0 to 1 (death = 0<
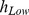
<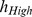 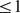 = perfect health). The drug is patent protected and offers a substantial improvement relative to existing therapies. Thus, it is assumed to be priced monopolistically. The individual's utility of health and consumption is taken as the function empirically estimated in [11]:
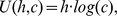
where *h* denotes health, and *c* denotes consumption measured in units of the minimum consumption required for existence (thus, *c* = 2, for example, means consumption twice that of the minimum consumption level). If we denote the maximal proportion of his consumption that the person is willing to give up in order to obtain the drug by *x*, we have:
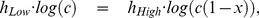
(2)where the left hand side is the utility without the drug, and the right hand side is the utility with the drug – better health, but only 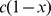 to consume. From this equality we obtain the maximal proportion *x*:
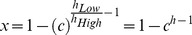
(3)where 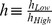 denotes the relative health with and without the drug.

The pharmaceutical company chooses the drug price 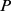 so as to maximize its profit. We make the simplifying, and typically fairly realistic, assumption that the pharmaceutical company's main cost is the development cost, which is a sunk cost at the time the drug is introduced, and that the production and marketing costs of the drug are insignificant for the determination of the cost of the drug (for some drugs, marketing costs may actually be very large, however, this is primarily so for “me too” drugs than for breakthrough drugs that typically receive a great deal of attention even with little marketing efforts [12]). We are focusing on the simplified case of a patient purchasing the drug directly from the pharmaceutical company, without an intermediary health provider, i.e. a “cash purchase”. Cash purchases represent about 30% of all drug sales [13]. While introducing an intermediary health provider greatly complicates the analysis (as the price in this case is typically composed of several different elements such as copayment, coinsurance, and deductibles), the more streamlined cash-purchase framework provides a clearer intuition of the main results, and it is therefore the framework adopted here.

**Figure 1:**
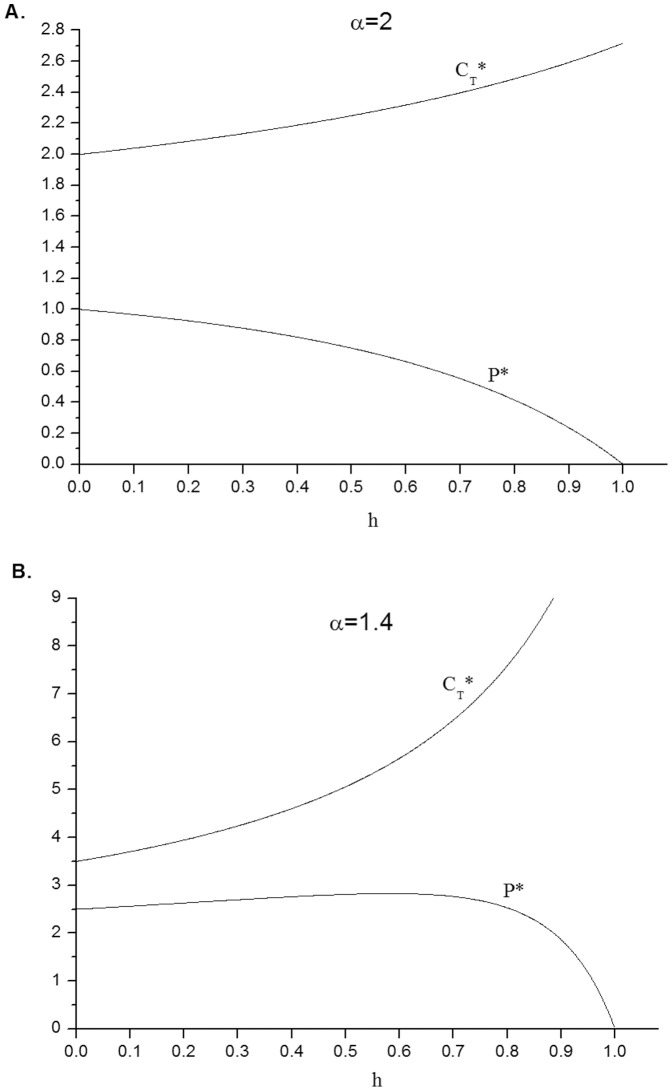
The optimal monopolistic drug price, P^*^, and the lower consumption threshold above which patients will purchase the drug, 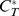, as a function of the relative health improvement, h (the lower h, the greater the health improvement). 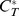
 is always increasing in h (see proof in footnote 7), i.e. the more dramatic the health improvement offered by the drug, the lower 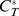, and the larger the proportion of patients who will use the drug. For typical values of 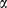, the consumption distribution Pareto exponent, P^*^ is monotonically decreasing in h, i.e. the more dramatic the health improvement the higher the drug price, as typically found empirically (see panel A). For low values of 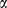, P^*^ may decrease with h over some range (see panel B).

Given a drug that provides a health improvement *h*, patients are willing to give-up up to a proportion *x* of their consumption, as given by eq.(3) for the drug. For a given drug price 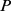, this implies that only patients with consumption exceeding a threshold consumption 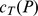 will buy the drug. 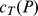 is given by:
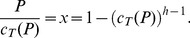
(4)For patients with 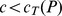 the fraction of their consumption that they would be required to pay for the drug, 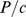, is too high relative to the alternative of living without the drug. Eq.(4) can be rewritten as:
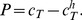
(5)The revenue of the pharmaceutical company is therefore:
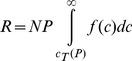
(6)where 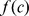 is the probability density function of the patient consumption distribution and *N* is the total number of patients. While eq.(6) is general, in order to obtain more concrete results one should assume a specific form for the consumption distribution. The most natural form to consider is the Pareto [14] distribution, which has been widely documented for both income and wealth for many different countries and economic conditions, and is given by:
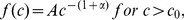
(7)where *A* and 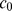 are positive constants, and 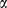 is the Pareto exponent (See [15] for a review of the literature on the Pareto distribution; we are interested in the consumption distribution, however, the income distribution, which is much more widely studied and documented, seems as a reasonable proxy for the consumption distribution). Empirical estimates of 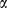 in Western countries range from 1.5 to 4, depending on the country and the year; see, for example, [16]. In the U.S. the estimated values of 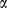 are typically in the range 1.5–2.0 [17], [18]. Employing the Pareto distribution (7) in (6) yields:
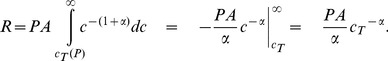
(8)As there is a one-to-one correspondence between the price *P* and the threshold consumption 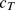 (see eq. 5), we can substitute 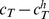 for *P*. This yields the pharmaceutical company's revenue as a function of 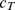:
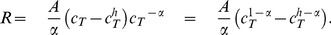
(9)Deriving this expression with respect to 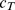 and equating to zero, we find the revenue maximizing value of 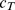, 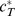:
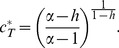
(10)Plugging this value in eq.(5) we obtain the equilibrium drug price,
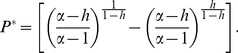
(11)The equilibrium values in equations (10) and (11) yield several interesting implications. First, note that 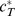 is monotonically increasing in 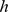 (see Appendix S1 in the supporting material). This means that the more substantial the health improvement provided by the drug, (i.e. the lower 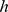), the lower 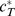, i.e. the drug will be priced such that it will be purchased by a larger part of the patient population. In contrast, 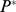 may either increase or decrease in 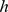, though for typical 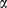 values 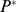 decreases with *h*, as one would intuitively expect (see Figure 1). This means that drugs that yield a more substantial health improvement (lower *h*) will have a higher price in equilibrium. This conforms with the empirical findings in [19]–[22]. In the next section we examine the implications of eqs.(10) and (11) for the debate about the regulation of drug prices.

## Implications for Price Regulation

Regulation of drug prices, which is applied in most countries excluding the U.S., obviously decreases the pharmaceutical company's revenues, while benefitting patients (at least those patients who suffer from a disease for which the drug has already been developed; one may argue that price regulation will prevent the development of new drugs and thus ultimately hurt patients – this is exactly the issue discussed in this section). The heated debate about price regulation is thus a debate about the relative weight of these two opposing effects of regulation. The framework developed in the previous section allows us to measure these effects in order to facilitate a quantitative discussion of the issue at hand.

It is important to note that price regulation is not a zero-sum setup, because a $1 decrease in revenue for the pharmaceutical company does not generally mean a $1 increase in consumer surplus. The consumer surplus can be much more (or less) than $1, depending on the exact shape of the demand function.

Consider a monopolistic drug which provides a health improvement *h* and is priced at price *P*, which is not necessarily the optimal monopolistic price. The pharmaceutical company's revenue is given by:
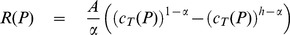
(12)where *c_T_(P)* is the threshold consumption given the price *P*, and is the solution to 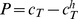 (see equations 5 and 9). Only patients with consumption exceeding this threshold will purchase the drug. This revenue is by definition lower than the maximal revenue at the optimal price P^*^ given by eq.(11), which is:
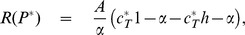
where 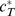 is given by eq.(10). The loss of revenue to the pharmaceutical company caused by regulating the price at *P* is thus:
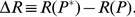
(13)


Regulating the price at 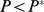 increases both the consumer surplus and the number of patients using the drug. A patient with consumption level *c* is willing to pay for the drug up to a proportion 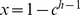 of his consumption, i.e. an amount of 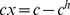 (see eq.(3)). Given a price *P*, patients with consumption above the threshold *c_T_(P)* will purchase the drug, and the aggregate consumer surplus is given by:
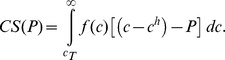
Substituting the Pareto distribution (7) for *f(c)*, and employing 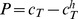 we obtain:
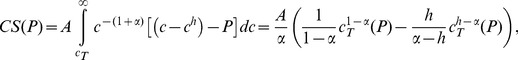
(14)The extra consumer surplus relative to the unregulated situation with the monopolistic price P^*^ is given by:
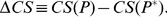
(15)


Figure 2 shows 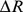 and 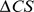 as a function of the price *P* for a typical drug with *h* = 0.5. With this value of *h*, and with a Pareto exponent of 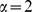, the optimal monopolistic price implied by eq.(11) is 0.75. Panel A shows the loss of revenue to the pharmaceutical company (in absolute terms) relative to the monopolistic price setup as a function of the price *P*. Note that by definition this function has a minimum at P^*^, where the revenue is maximal (and thus the loss of revenue is minimal). Hence, moderate changes in *P* around P^*^ have only a second-order effect on the revenues. In contrast, the effect on consumer surplus is first-order, see Panel B. While both 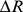 and 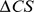 shown in Figure 2 are in units of the minimal consumption times the Pareto constant *A* (see eq.(7)), they are both in the same units. This implies that imposing a price of, for example, *P* = 0.6, leads to a consumer surplus which is roughly 10 times as large as the decrease in revenues.

**Figure 2:**
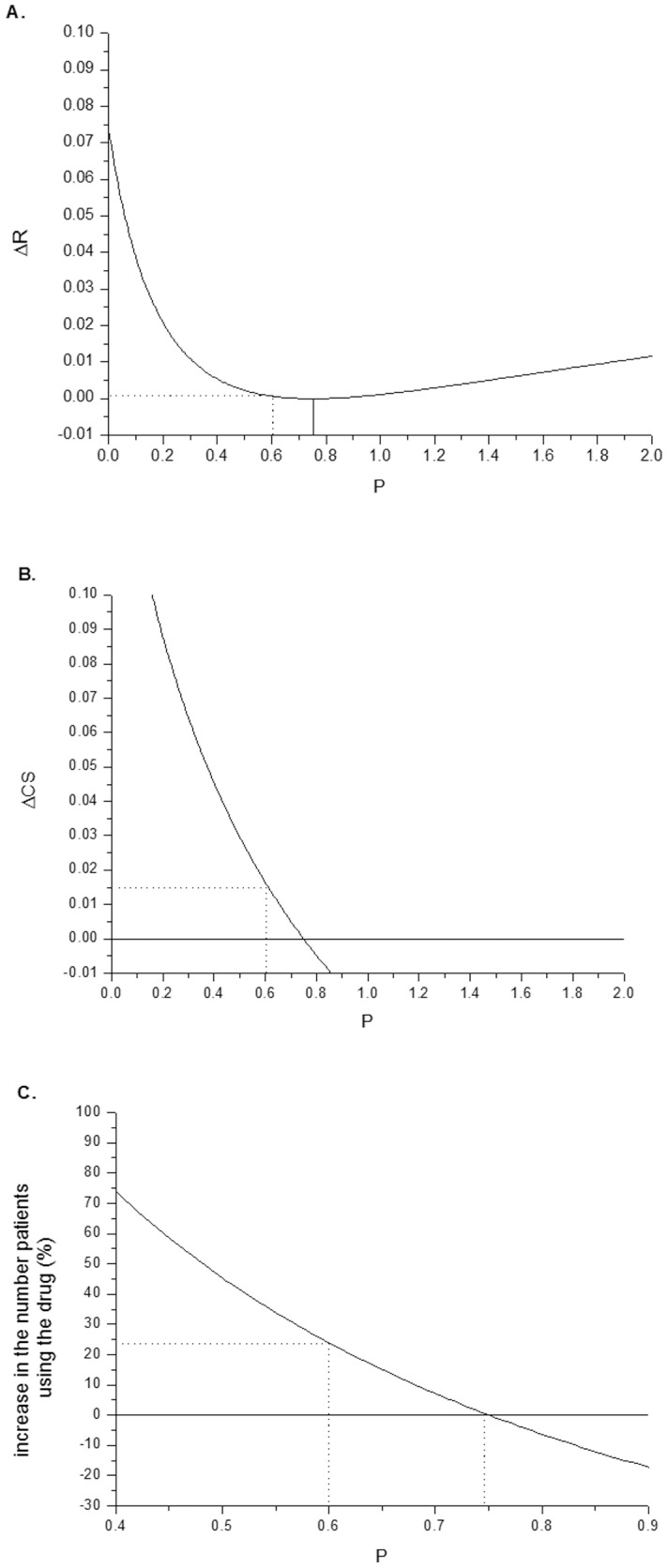
The effect of the drug price, P, on the loss of revenues (in absolute terms) of the pharmaceutical company (panel A), the consumer surplus (panel B), and the number of patients using the drug (panel C). The case shown is for a health improvement h = 0.5 and a Pareto exponent 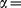
2. For these typical parameters, the optimal monopolistic price is P^*^ = 0.75 (in units of the minimum consumption level). Placing a price cap of P = 0.6 dramatically increases the consumer surplus and the number of patients using the drug, while having only a marginal effect on the revenues of the pharmaceutical company.

Given a certain price *P*, the number of patients using the drug is:
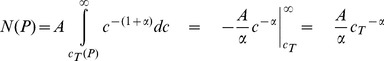
(16)where the lower threshold *c_T_* is the solution to 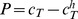. In the unregulated monopolistic case, the number of patients using the drug is 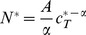. Panel C of Figure 2 shows the relative change, in percent, of the number of patients using the drug as a function of the price *P*: 
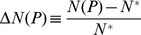
. The figure shows that imposing a price cap of 0.6 increases the number of patients using the drug by about 25%, relative to the unregulated situation.

Figure 2 implies that a small change in price relative to the monopolistic price has a first-order effect on consumer surplus and the number of patients using the drug, but only a second-order effect on revenues. From this perspective it is clear that some amount of price regulation is socially desirable. Of course, the practical question is how much regulation is not too much? If a price cap is set too low, this may have a drastic influence on revenues, stifling all R&D incentives for the pharmaceutical companies. Tables 1 and 2 present some quantitative results regarding this issue. Table 1 reports the effects of imposing a price cap which is 20% lower than the monopolistic price (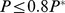), for various different drugs (different values of *h*). This amount of regulation leads to a decrease in revenues of only about 1%, but to an increase in surplus of about 10%. The magnitude of 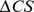 is about 25 times the magnitude of 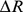. The regulation leads to an increase of about 23% in the number of patients using the drug.

**Table 1:** The effects of price regulation in the form 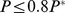, i.e. the price is set 20% lower than the optimal monopolistic price.

(1)	(2)	(3)	(4)	(5)	(6)	(7)	(8)
*h*	P^*^	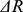	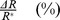	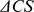	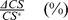	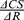	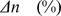
0.10	0.97	0.0014	1.18	0.0258	10.83	18.90	23.53
0.20	0.93	0.0012	1.12	0.0237	10.53	19.91	23.61
0.30	0.88	0.0010	1.06	0.0215	10.22	21.04	23.68
0.40	0.82	0.0009	1.00	0.0191	9.89	22.30	23.76
0.50	0.75	0.0007	0.94	0.0165	9.53	23.75	23.83
0.60	0.66	0.0005	0.88	0.0137	9.16	25.38	23.91
0.70	0.55	0.0004	0.82	0.0107	8.77	27.28	23.98
0.80	0.41	0.0003	0.75	0.0075	8.35	29.50	24.05
0.90	0.24	0.0001	0.69	0.0039	7.90	32.11	24.13

This price constraint lowers revenues by only 0.69%–1.18% relative to the monopolistic revenues, depending on *h*, the benefit provided by the drug (4). The consumer surplus is increased by 7.9%–10.8% relative to the unregulated case (6). The increase in consumer surplus is about twenty-fold to thirty-fold the decrease in revenues (7), and the number of patients using the drug increases by about 23% relative to the unregulated case.

**Table 2:** The effects of price regulation in the form 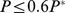, i.e. the price is set 40% lower than the optimal monopolistic price.

(1)	(2)	(3)	(4)	(5)	(6)	(7)	(8)
*h*	P^*^	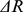	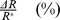	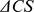	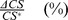	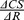	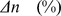
0.10	0.97	0.0069	5.98	0.0581	24.38	8.37	56.71
0.20	0.93	0.0061	5.70	0.0534	23.73	8.79	57.16
0.30	0.88	0.0052	5.42	0.0484	23.04	9.25	57.64
0.40	0.82	0.0044	5.13	0.0430	22.31	9.78	58.10
0.50	0.75	0.0036	4.85	0.0372	21.54	10.37	58.60
0.60	0.66	0.0028	4.55	0.0310	20.71	11.05	59.09
0.70	0.55	0.0020	4.25	0.0242	19.84	11.84	59.57
0.80	0.41	0.0013	3.95	0.0169	18.90	12.75	60.08
0.90	0.24	0.0006	3.65	0.0088	17.91	13.83	60.59

In this case the decrease in revenues is 3%–6% (see column 4), much more substantial relative to the case shown in Table 1. While the increase in consumer surplus is also larger (6), the ratio between the consumer surplus increase and the revenue decrease is lower than in the case of 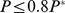 (compare column (7) with column (7) in Table 1).

Table 2 reports the same analysis, but this time for the case of a price cap that is 40% lower than the monopolistic price (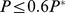). In this case the loss in revenues of the pharmaceutical company is significant, and can reach 6%. While the consumer surplus also increases, the ratio 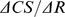 is lower than in the case shown in Table 1, and is now only about 10. Thus, it seems that this type of price cap is “going too far”, in the sense that the costs to the pharmaceutical companies may be too severe. In any case, the optimal monopolistic price formula, given by eq.(11), provides a useful benchmark as a basis for price regulation.

## Conclusions

Healthcare expenditures in the U.S. are estimated at $2.8 trillion, representing about 17% of GDP [23]. The percentage of health expenditures as a fraction of GDP is increasing over time, not only in the U.S., but worldwide [24]. It is thus not surprising that economic discussion of healthcare has become a central issue for policy and academic research.

In this paper we focus on the debate about drug price regulation. While critics of the pharmaceutical industry argue that pharmaceutical companies exploit monopolistic power granted by patent protection to make “unreasonable” profits at the expense of patients, proponents of this industry claim that high drug prices are required to sustain the very large R&D expenditures in pharmaceuticals. An in-depth discussion of this important issue requires a quantitative framework of analysis. This paper suggests such a framework.

The analysis is conducted in the standard expected utility framework, where well-being, or “utility”, is a function of both health and wealth [8]–[10]. We employ recent empirical findings in [11] about the shape of the utility function of health and wealth to formulate a model of the optimal monopolistic pricing of breakthrough drugs. This optimal monopolistic price then serves as the basis for price regulation, i.e. the regulated price is determined in terms of the monopolistic price. Thus, the model provides a theoretical foundation and benchmark for setting price caps. The model allows us to quantify the costs and benefits of drug price regulation. We find that mild price regulation can substantially increase consumer surplus and the number of patients using the drug, while having only a second-order effect of the revenues of the pharmaceutical companies. For example, setting the price cap at 20% lower than the optimal monopolistic price increases the consumer surplus by about 10%, and increases the number of patients using the drug by about 23%. This increase in the number of users almost completely offsets the adverse effect of the price regulation from the perspective of the pharmaceutical company – its revenues decrease by only about 1%. However, more aggressive price regulation leads to a substantial revenue reduction, and may stifle innovation. The price caps in OECD countries, which are up to 67% lower than the U.S. unregulated prices, lead to a lower ratio between the consumer surplus and the loss of revenue for the pharmaceutical company, and thus certainly seem excessive. There seems to be a “golden path” of mild regulation that on the one hand greatly improves patient welfare, and on the other hand does not stifle the pharmaceutical industry and the important economic incentive for drug innovation.

## Supporting Information

Appendix S1
**Proof that** 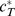 **is monotonically increasing in** 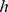 **.**
(DOCX)
