# Supplementary material for: The Pricing of Breakthrough Drugs: Theory and Policy Implications
Source: PLoS One. 2014 Nov 25;9(11):e113894. doi: 10.1371/journal.pone.0113894 (PMC4244177; doi:10.1371/journal.pone.0113894)
Supplement: Appendix S1 — Proof that is monotonically increasing in . (DOCX) [file pone.0113894.s001.docx]

**Appendix S1**

$c_{T}^{*}$ can be written as $c_{T}^{*}=f(h)^{g(h)}$, where $f(h)\equiv\frac{\alpha-h}{\alpha-1}$and $g(h)\equiv\frac{1}{1-h}$. Recall that $\frac{\partial}{\partial h}\left[f(h)^{g(h)}\right]=f(h)^{g(h)}\left[g'(h)\ln(f(h))+(g(h)/f(h))f'(h)\right]$. With *f(h)* and *g(h)* as defined above we have $f'(h)=\frac{1}{1-\alpha}$and $g'(h)=\frac{1}{\left(1-h\right)^{2}}$, and therefore:

$\frac{\partial c_{T}^{*}}{\partial h}=c_{T}^{*}\left(\frac{1}{1-h}\right)\left[\frac{1}{1-h}\ln\left(\frac{\alpha-h}{\alpha-1}\right) - \frac{1}{\alpha-h}\right]$. To see that the expression in the preceding square brackets is positive, note that the natural logarithm can be expressed as: $\ln(z)=2\sum_{n=0}^{\infty} \frac{1}{\left(2n+1\right)}\left(\frac{z-1}{z+1}\right)^{2n+1}$. Here we have $z=\frac{\alpha-h}{\alpha-1}$, and $\frac{z-1}{z+1}=\frac{1-h}{2\alpha-h-1}$, which is positive because $\alpha>1>h$. This implies that $\ln\left(\frac{\alpha-h}{\alpha-1}\right)>2\left(\frac{1-h}{2\alpha-h-1}\right)$, because the right hand side takes only the term *n*=0 of the expansion, and ignores all the other terms with *n*>1, which are all positive. Thus, we have: $\frac{\partial c_{T}^{*}}{\partial h}>c_{T}^{*}\left(\frac{1}{1-h}\right)\left[\frac{1}{1-h}2\left(\frac{1-h}{2\alpha-h-1}\right) - \frac{1}{\alpha-h}\right]=c_{T}^{*}\frac{1}{\left(2\alpha-h-1\right)\left(\alpha-h\right)}>0$, which proves that $c_{T}^{*}$ increases with *h*.
